# Enabling pathways to health equity: developing a framework for implementing social capital in practice

**DOI:** 10.1186/1471-2458-13-517

**Published:** 2013-05-29

**Authors:** Christine Putland, Fran Baum, Anna Ziersch, Kathy Arthurson, Dorota Pomagalska

**Affiliations:** 1 Southgate Institute for Health, Society and Equity, Flinders University, Bedford Park, South Australia

**Keywords:** Social capital, Health inequities, Community development, Policy and practice, Health promotion

## Abstract

**Background:**

Mounting evidence linking aspects of social capital to health and wellbeing outcomes, in particular to reducing health inequities, has led to intense interest in social capital theory within public health in recent decades. As a result, governments internationally are designing interventions to improve health and wellbeing by addressing levels of social capital in communities. The application of theory to practice is uneven, however, reflecting differing views on the pathways between social capital and health, and divergent theories about social capital itself. Unreliable implementation may restrict the potential to contribute to health equity by this means, yet to date there has been limited investigation of how the theory is interpreted at the level of policy and then translated into practice.

**Methods:**

The paper outlines a collaborative research project designed to address this knowledge deficit in order to inform more effective implementation. Undertaken in partnership with government departments, the study explored the application of social capital theory in programs designed to promote health and wellbeing in Adelaide, South Australia. It comprised three case studies of community-based practice, employing qualitative interviews and focus groups with community participants, practitioners, program managers and policy makers, to examine the ways in which the concept was interpreted and operationalized and identify the factors influencing success. These key lessons informed the development of practical resources comprising a guide for practitioners and briefing for policy makers.

**Results:**

Overall the study showed that effective community projects can contribute to population health and wellbeing and reducing health inequities. Of specific relevance to this paper, however, is the finding that community projects rely for their effectiveness on a broader commitment expressed through policies and frameworks at the highest level of government decision making. In particular this relationship requires long term vision, endorsement for cross-sectoral work, well-developed relationships and theoretical and practical knowledge.

**Conclusions:**

Attention to the practical application of social capital theory shows that community projects require structural support in their efforts to improve health and wellbeing and reduce health inequities. Sound community development techniques are essential but do not operate independently from frameworks and policies at the highest levels of government. Recognition of the interdependence of policy and practice will enable government to achieve these goals more effectively.

## Background

There is growing evidence that aspects of social capital produce benefits in terms of health and wellbeing and assist in reducing health inequities. This knowledge has been amplified by the WHO Commission on the Social Determinants of Health [1,2].

Social capital theory holds that social connections embody value to both individuals and society as a whole, therefore social networks and relationships between people are important resources. Beyond this central idea, competing theories about social capital highlight its complexity in terms of different kinds of resources operating on different levels, with sometimes contradictory effects [3,4]. This complexity has not prevented considerable interest in social capital theory within public health during the past few decades, neither has it slowed the development of strategies based on social capital theory to address health inequities at the community level. These approaches draw on growing evidence that social capital, in the context of broad policy approaches, can have significant impacts on health and wellbeing [5]. The application of theory to practice tends to be uneven, however, largely reflecting the difficulty of translating complex, abstract ideas into coherent programs with clearly defined health outcomes. If the potential value of social capital as a way of working is to be realised there is a need to focus more directly on this process of how theory is successfully or unsuccessfully translated into practice.

This paper reports on a study which aimed, firstly, to develop an understanding of how social capital theory informs government programs designed to reduce health inequities, and then sought to explore how this knowledge can be used to enable its effective application. It describes the background to the study and then outlines the design of the collaborative research process with government partners which led to practical outcomes in the form of guides to support community-based practice in initiatives designed to reduce health inequities. The paper proceeds to show how analysis of the findings in partnership with a representative group of policy makers and senior managers responsible for implementing the programs identified key lessons about the factors enabling these pathways.

### Social capital theory and health

Resources associated with social and economic status and their availability to individuals and groups have long been linked to health and wellbeing. According to the Commission on the Social Determinants of Health [1] a complex web of social and economic processes is responsible for health inequities, or systematic differences in health status between socio-economic, gender, race and geographic groupings. Strategies to improve health and wellbeing involve action at a range of levels: from healthy public policies to create the conditions for health, shaping opportunities and access to resources for individuals and communities, to supportive local environments which encourage and strengthen community action and develop individual skills. These aspects are characterised by the Commission for the Social Determinants of Health, respectively, as ‘macro’ , ‘meso’ and ‘micro’ level policy interventions that are necessary to reduce health inequities [1]. Social capital theory is commonly associated with ‘micro’ level community-based interventions, although theorists vary in emphasis, two of the most influential being Putnam [6] and Bourdieu [7].

Putnam’s concern with social cohesion and efficiency leads to a definition of social capital as ‘the features of social organisation such as networks, norms and social trust that facilitate coordination and cooperation for mutual benefit’ [6] (p 67). Bourdieu [7] stresses the value of social capital as a resource enabling individual access to a range of other capitals, including economic and cultural capital, through the mobilisation and leverage of social networks. Although both interpretations rely on relational (membership in social organisations and networks) and material (the resources that flow from being part of the group) elements, they have different implications in terms of devising pathways to health and healthy equity. For Putnam, the stocks of norms and trust within communities are assets that may be turned into health outcomes by facilitating the modelling of positive behaviours, regulating and controlling negative behaviours and supporting collective action for shared benefit. Through such social processes as ‘collective socialisation’ , ‘informal social control’ and ‘collective efficacy’ , features of social capital are thought to lead to a more cohesive, productive and healthier society [8]. Bourdieu [7] by comparison is concerned with power and the ways in which advantage and disadvantage are reproduced and maintained within socio-economic groups through such networks. Unlike Putnam, for whom individual behaviour contributes to creating more coherent and safer societies, Bourdieu engages with the relationship between social capital and social inequity, focusing on how society can support individuals through enabling more equal access to resources [9].

### Evidence for the linkages between social capital and health

Evidence for the links to health and wellbeing outcomes has been growing in recent decades at the micro, meso and macro levels [1,10-13]; although the issue of measurement remains controversial. Most relevant to this discussion are findings in relation to community level interventions, consistently demonstrating that networks can be an important resource for health, with strong social supports linked to improved health outcomes and reduced mortality rates. Distinctions have been made between three different types of social networks and the sorts of social capital they provide (bonding, bridging and linking). Close personal networks producing trust, reciprocity and belonging have been found to create an effective buffer from stress [13], often referred to as ‘bonding social capital’ [14]; The effect is thought to be strengthened by ‘bridging’ (between different social groupings) and ‘linking’ (vertical connections) social capital; networks enabling opportunities for democratic and civic involvement and linking to people and institutions in power that may be leveraged against material or financial gain [5,9]. At the neighbourhood level there is some evidence that areas with higher social capital have better health outcomes [15-17].

### Mobilising social capital through public policy

The growing evidence for features of social capital as determinants of health and wellbeing has simultaneously highlighted its potential as a pathway to other determinants, and therefore its relevance to a range of policy sectors including welfare, education, families and communities, employment, housing, urban development and planning and justice. While securing its broad public policy appeal, this diversity has arguably added to the controversy about definition, measurement, means of mobilisation and expected outcomes [3,4].

As Castiglione et al. [18] (p 6) note, ‘the attractiveness of social capital for policy making lies both in the generally positive connotation that is often attributed to social capital’s presence in society, and in its causal role in the production of social and individual goods.’ Thus although interest in social capital waxes and wanes, its associated ideas continue to be cited as justification for government programs in Australia and elsewhere as a means of improving the lives of individuals and whole communities. Less evident, however, is how it is interpreted and implemented in practice, yet this is clearly crucial for its promise to be realised. This paper reports on a study which contributed to redressing this imbalance by examining the question of how social capital is interpreted and applied in policies and programs designed to improve health from the perspective of policy actors and community practitioners.

## Methods

As an example of applied policy research, the broader study on which this paper is based reflected the dual purposes of seeking to understand how social capital theory is employed in policy and practice, and using this knowledge for the purpose of informing implementation [19]. It drew lessons from a series of community case studies demonstrating the application of social capital in a ‘real world’ setting [20]. As is typical in case study design, these were based on multiple sources of data [20], including direct accounts from individuals responsible for developing programs and policies as well as those implementing the latter in the form of community projects. This paper derives selectively from the study findings, focusing specifically on how these policy actors and community practitioners conceptualised social capital as part of their work and used it to promote health in an equitable way. This is an important source of information because the translation of complex theories into practice is not always explicit or transparent in project documentation.

The study, undertaken between 2004 and 2007, involved a staged process of data collection punctuated at strategic points by input from a Reference Group to inform subsequent stages. Knowledge transfer was supported by collaboration with the three state and local government Industry Partners (IPs) who were party to the Australian Research Council Linkage grant: SA Department of Health, Arts SA and City of Onkaparinga. Representatives of these partner organisations were co-investigators on the study, bringing to it their interpretation of the emerging case study data from an organisational perspective and joining with other government representatives to form the Reference Group which guided the research process and practical outcome. The rigour of the design relied on this negotiated iteration of ideas and research process in collaboration with research partners [21]. The findings at each stage were not only integral to the way in which the research process unfolded but also to the method of disseminating knowledge from the study to the field of practice.

### Case studies

Discussions with the IPs during the preparation of the research grant application led to the identification of programs employing the principles and broad terminology of social capital and the selection of three specific cases of community-based projects aimed at improving health and well-being. The case study approach to empirical research, designed to capture a ‘phenomenon in context’ based on a range of data sources, is well-suited to understanding complex policy implementation and programs [20] (p179). Multiple cases were chosen to facilitate examination of the research focus in the context of different communities and locations [20]. Selection of case studies was primarily opportunistic, beginning with a broad scan of state and local government websites in order to ascertain those programs that were likely to be relevant. From this initial scan of publicly available documentation and discussions with Industry Partners we identified potential case studies which were informed by principles commonly associated with social capital such as ‘social networks’ , ‘resources’ , ‘trust’ , ‘equity’ , and ‘community belonging’ , as well as more current policy terms such as ‘social inclusion’ and ‘community capacity building’. Three particular initiatives in this group were identified in consultation with IPs as providing examples of social capital in practice which, while originating from different government agencies and sectors, had several features in common. Each was located in a metropolitan area experiencing high levels of unemployment, while two out of three of the areas were also undergoing major urban renewal with associated community disruption. The initiatives were managed by inter-sectoral partnerships and each comprised a number of smaller projects employing creative activities – visual and public art, music, crafts and gardening – as vehicles to engage community participants. The similar profiles, summarised in Table 1, reflect the common purpose of the government programs designed to address health through social capital.

**Table 1:** Summary of case study projects

**‘Yangara reserve’ (O’Sullivan’s Beach, Southern suburbs)**	
*Partners:*	Local government, non-government, community
*Main focus:*	Redevelopment of outdoor space
*Context:*	Isolated from neighbouring areas; Indigenous population & significance of reserves; high unemployment; poor access to public transport
*Objectives:*	Community engagement; connect community groups; redevelop Reserve; sense of community pride & ownership; attract external funding
**‘Parks Helix’ (‘The Parks’ estate, Woodville, western suburbs)**	
*Partners:*	Local government, State government (Arts, Health, Education), Community Sector (state and local funding), Private Industry (housing developer)
*Main focus:*	Art and cultural program linked to redevelopment
*Context:*	Major urban renewal causing community upheaval; concentrated social disadvantage; issues of crime & safety
*Objectives:*	Social inclusion; community capacity building; formal & informal networks; individual & community wellbeing; partnerships among services; raise profile of arts & culture
**‘KBA community capacity building’ (Kilburn Blair Athol, northern suburbs)**	
*Partners:*	State government (Health, Housing)
*Main focus:*	Series of projects involving different population groups
*Context:*	Urban regeneration; community consultation identified multiple issues; community development workers employed
*Objectives:*	Health & wellbeing; new & existing networks; sharing resources & skills; social outlets; projects addressing issues of drugs, alcohol, violence

A purposive sampling strategy was adopted to gain a diversity of views from a range of perspectives within each of these sites. Community participants, local staff, artists and volunteers as well as policy actors and senior managers, were selected on the basis of their detailed knowledge of the phenomenon in question [22]. This was supplemented by opportunistic sampling to take advantage of opportunities to recruit additional participants in the course of the study [22]. Recruitment ceased when a point of saturation was reached and it became clear that no new information was emerging and the existing data would support the intended analysis [22].

#### Data collection methods

The iterative, staged process and range of informants called for multiple qualitative methods to generate a rich understanding of meaning, process and interpretation of experiences, concepts and theories [21,22]. The methods included focus groups, in-depth interviews, observation and document analysis. Focus groups were employed to bring together community members, volunteers and local staff in the familiar setting of the community project to discuss their views on the shared experiences of participating in the program [22]. Interviews with individual staff, senior managers and policy actors provided first-hand accounts and observations and the opportunity to explore ideas and concepts [21]. These were supplemented by researcher observation and document analysis to provide a context for the group and individual accounts [21]. Bearing in mind that this paper draws on selected data, the components of the study are outlined below and the sources reported on in this paper are indicated.

1. Project case studies

Data collection comprised in-depth individual interviews with 20 staff across the cases including program coordinators, service/agency managers, artists and community development workers; 15 focus groups attended by over 100 community participants, volunteers and local staff; guided observation of program activities; and, document analysis including project evaluation reports, journals and art works (visual, text, public art). Study participants were recruited with the assistance of the IP representatives and using attendance records to identify community members in local projects and issue invitations to attend through the project networks. Focus groups canvassed a wide range of themes including how participants became involved, how the project worked on a daily basis and the perceived personal and community benefits. These data are not the focus of this paper. Interviews with individual staff were designed to capture a snapshot of practice at a particular point in time and covered detailed questions related to community engagement, social capital, project processes and outcomes, barriers and facilitating factors in participation. This paper draws directly on these interview data.

2. Senior manager and policy actor interviews

a. Face-to-face in-depth interviews were conducted with 22 individual senior public servants within the state and local government departments and divisions responsible for the programs from which the case studies were drawn. A ‘criterion sampling’ approach which included all those who met the relevant criteria was adopted in relation to this group of participants [22]. Interviewees were recruited on the basis of their position in the government department or agency in charge of one of the projects that were the subject of the study. In each case they were responsible for funding the community programs or for drafting the broad policies which shaped their implementation. This list was supplemented by people in similar positions in the Justice Department which had been involved in the original funding application but which ultimately was unable to provide a relevant case example based on social capital theory. In addition, two local government elected members from the City of Onkaparinga were recruited based on their pivotal role in the development of the case study project, ensuring its carriage through Council funding processes. Each participant was approached individually and all agreed to be interviewed. They provided general information about the level and type of use of social capital theory in government programs and the reasons for and against its application, as well as an overview of its interpretation in their specific areas of responsibility. These senior level public servants are referred to below as ‘Administrators’.

a. Throughout the study a series of workshops and ‘Think Tanks’ were held at strategic points, where findings from each stage of the research were presented and discussed with the Reference Group members. Their feedback guided subsequent research stages as well as the final form and content of the guides comprising the practical outcome of the research.

Study participants were provided with information about the research project and assurances of confidentiality and anonymity and completed consent forms prior to their involvement in the study, based on ethics guidelines and approval received from the Flinders University Social and Behavioural Research Ethics Committee (Project 3477, granted 11 April 2006). The study was conducted in compliance with the Helsinki Declaration.

#### Data analysis

Data were summarised and analysed incrementally throughout, led by the research team with regular feedback on drafts and reports from Reference Group members. Transcriptions of recorded interviews and written notes from focus groups together with documentation were compiled and presented to the quarterly meetings of the Reference Group. This iterative process served as triangulation in order to develop a complex picture of the phenomenon being studied [22]. In the context of applied policy research, a broad framework approach was necessary to meet specific information needs and provide practical outcomes to inform the implementation of social capital theory in practice [22]. The qualitative and discursive form of the data called for broad thematic analysis and identification of standout points in relation to the research objectives [22]. Coding processes employed can be described as ‘template’ in which codes are determined from research questions and an initial reading then text segments identified, and ‘immersion’ emphasising researcher interpretation of meaning and insight [21]. In addition to the sound audit trail and the triangulation achieved by member-checking with the reference group mentioned above, rigour was also ensured with coding by two or more researchers to check the consistency of interpretation and any differences being discussed and resolved within the wider research team. This study adheres to the RATS guidelines on qualitative research.

Reflecting the focus of this paper, the discussion of Results that follows draws exclusively on data from staff interviews in component 1 above and interviews with senior managers and policy actors in component 2. Quotations from interviewees are used to illustrate the themes and highlight both ‘typical’ and ‘atypical’ views. To distinguish the source, staff are referred to as ‘practitioners’ (includes artists and community workers) or ‘managers’ (includes agency managers and program/project coordinators), while senior managers and policy actors are referred to as ‘Administrators’.

## Results and discussion

Below we summarise the lessons that emerged from the study and show how they informed the shape of the practical research outcome. Reflecting the study aims and design, the results are presented in three parts:

1. Social capital in policies and programs.

2. Factors influencing effective implementation at the community level.

3. Advocacy for a social capital approach.

### Social capital in policies and programs

Consultation with senior managers and those responsible for developing policies and programs provided insights about how the concept of social capital was understood and interpreted in government programs, and the issues that arose in this process. Here we examined the ways in which social capital was defined by these ‘Administrators’, their perceptions of the applicability of social capital to practical projects and the perceived value of social capital to health and well-being.

#### Defining social capital?

The informants in this study indicated that social capital was hard to define despite its familiarity. On the one hand the broad ideas associated with social capital have become common place and also part of public policy thinking:

I think it [social capital] is a little more deeply embedded into our community and society’s psyche now…it’s not so much a catch- phrase, as something we take consideration of in the same way we look at economic impacts or environmental impacts. (Administrator)

Aspects of social capital formation such as ‘social connection’ , ‘cohesion’ , ‘networks’ and ‘trust’ were referred to in describing the concept, although these were not attributed to any particular theory. On the other hand, beyond a general understanding, the debates were regarded as fairly inaccessible to a non-academic audience, citing confusing terms and a lack of clarity about what it means in practice as causes:

It [social capital] is one of these terms that has achieved a certain prominence but not necessarily a level of understanding of what it is and how it’s measured. (Administrator)

The need for clear and simple definitions was repeatedly noted:

I think people have moved away from the original definition and it’s kind of woolly now…I don’t think people really know what it is they’re talking about apart from something warm and fuzzy. (Administrator)

You break it down and say, ‘okay, what does all this mean?’ And you bring it down to concepts that people understand… (Administrator)

#### A ‘social capital type of approach’

In discussions of what a ‘social capital approach’ entailed in practice respondents clarified that it is strongly associated with working in local communities, along with a number of apparently comparable approaches:

There are so many of those buzz words at the moment that are out there and it’s so easy to trip them off your tongue…community development…social inclusion… community engagement… (Administrator)

Community development and capacity building strategies were regarded as the operational basis of a social capital approach, the practical technique giving expression to the theory:

…I go back to good old fashioned community development … (Administrator)

In some form community capacity building …has occurred for thirty or forty years… (Administrator)

I believe good solid community development builds social capital, so projects or approaches with community development principles which are looking usually for a solid, often a tangible outcome… (Administrator)

The concept of social inclusion was acknowledged to be closely related to social capital and for the most part concerned with similar issues:

Certainly they’re [social capital and social inclusion] related because if you’re not included or you’re excluded for reasons of poverty, unemployment, you might be seen as being not part of the norm… (Administrator)

Despite some uncertainty about the differences, for many the concept of social inclusion was more self-evident and easier to define:

Social inclusion…it’s a program of government designed to ensure that more people in the community are included in the full range of benefits – of community benefits and resources that the community has access to… it acknowledges that there are individuals and groups in the community who are less likely to be participants in community life. And it says we are interested in trying to ensure that programs, government undertakings, all kinds of things – community life in general – are more accessible to a full range of people. (Administrator)

This finding can partly be explained by the more explicit use of the term social inclusion at the State level where it had been a policy driver from the time of the election of the Labor Government in 2002 [23]:

In South Australia within government we talk more of social inclusion because of our Social Inclusion Initiative, and that was the direction that the Labor Government in 2002 took the debate about social capital…but people still use the term [social capital] across government. (Administrator)

In attempts to distinguish between the approaches, social capital tended to be linked to a concern with ‘community’ , ‘connection’ , and social relationships, while social inclusion was thought to be part of a more extensive system: the cultural, social and economic processes operating at the level of individuals, organisations and more broadly. In one case, community capacity building was thought to be more directly concerned with social change:

…[when I think of social capital] I think of what are the types of things that are in place for the community to have the capacity to problem-solve, to address issues, to work together, to support communities…[whereas] community capacity building might also include leaders who might make change and I don’t think that’s necessarily something that I would see as part …of social capital. (Administrator)

These kinds of distinctions suggest an understanding of social capital that draws mainly on the idea of connection and cohesion aligned with Putnam’s [6] view. Overall, however, the discussions indicated that many struggled to understand the different theories in any detail.

#### Perceived value of social capital for health and wellbeing

Despite the lack of detailed knowledge the about a social capital approach, there was a high level of acceptance of the potential health and wellbeing benefits of social capital amongst informants. Its value was generally described in the context of a range of social and economic factors:

So from my perspective if you are healthy, you are… you have stable accommodation, you have good community connections, you have access to employment, even if you choose not to exercise that access, and you have the trappings that enable you to live a comfortable life that is what you’re aspiring to. (Administrator)

The general impression, however, was that informants in this study considered themselves more favourably disposed towards the theory than many of their colleagues and they expressed doubt about the wide acceptance of the practical pathways to health and wellbeing. Notwithstanding good intentions, the fact that results are not always immediate and some effects take years before they can be clearly assessed meant that many remained unclear about the process:

I think they [social capital initiatives] are very beneficial, but [we need] understanding up front of what are they’re actually trying to deliver. What is the objective of actually implementing [them] and understanding how has it worked out? (Administrator)

The sporadic application of a ‘social capital approach’ in public health was attributed to this lack of clarity coupled with a narrow definition of ‘health’ – involving medical intervention and reduction of specified medical risk factors – seen to dominate public policy thinking. Social capital, by contrast, is more readily understood in terms of a general sense of wellbeing and the underlying conditions associated with health creation including social support and socio-economic resources. The perceived ‘health crisis’ revolving around funding for acute care was thought to have directed policy attention away from such strategies for long term sustainability and towards immediate ‘crisis management’ solutions.

I think government, and our colleague agencies, education, transport, whatever, I think they struggle with seeing the broadness of the concept [of health]…. (Administrator)

It does essentially come back to a central area… how the dynamics changed essentially in the hospital setting [and the need to] relieve pressure in this area? (Administrator)

So when we are reporting to Treasury they would be asking, are they [community programs] achieving what they were intended to achieve? Are they delivering in terms of relieving the hospital pressure? (Administrator)

Crucially, appreciation of the value of social capital at the most senior levels was recognised as a significant factor in implementing a social capital approach:

If major decisions are made at the top level it will affect the work that happens on the ground. (Administrator)

In this light, there was a perceived need for better communication of evidence for the benefits of social capital to those in decision making roles. The point was made that despite a commitment by individuals, administrators and policy makers battle competing demands and priorities:

…at the end of the day, we’re also running complex administrative units and we’ve got budgets to balance, and so many competing priorities as well. Sometimes it [takes] a miracle [for] good things to happen, but it’s not for a lack of good people, [just] for competition amongst priorities. (Administrator)

Convincing evidence supported by accessible theorising was deemed necessary to enable the effective translation of social capital theory to policy and programs.

### Factors influencing effective implementation at the community level

As described above, informants in the case studies included program managers at the local agency level, community development workers and artists all of whom provided insights into the ways in which social capital is put into practice. Together with comments from ‘Administrators’ , the views of these ‘Managers’ and ‘Practitioners’ highlighted a number of factors that were thought to determine effective implementation of these programs.

#### Community development experience

As highlighted above, community development practice was regarded as the operational arm of social capital. Standard practice in this field includes planning and consultation, building on community strengths and assets, effective vehicles for engaging participants and strong relationships with local people based on trust and respect. These activities were seen as relying on in-depth experience and high levels of skill:

It has been very successful but what I would like people to understand is it just doesn’t happen. It needs to be facilitated with very clear boundaries. (Manager)

We all know that behind the scenes there is a lot of hard work. (Practitioner)

The one thing that is very important in community-based work really often comes down to the personal relationship between key workers and those that become involved. (Manager)

Recognition on the part of employing agencies and organisations of the range and diversity of skills involved in community development practice at this level was seen as important to successful outcomes.

#### Organisational commitment

Participants felt that the kinds of fundamental changes that are implied in a social capital approach to health equity meant that they are not achievable through short term projects but require sustained engagement.

You can’t think that you are going to build any type of community in ten weeks… most people are just starting in ten weeks to feel comfortable and then the money stops. (Practitioner)

With community development programs… you are looking at anything from 2 to 3 years to begin to see outcomes… (Who is this from?)

…and some of the benefits do not appear until 10 or more years later… (Practitioner)

Not only did community development rely on a long term commitment to the underlying values, but it also required a flexible approach in order to respond to the expressed needs of the community. This was regarded as easier for some agencies than others:

…we’re [in local government] very lucky in that …we can be much more flexible than people like the Housing Trust or … Health… because we don’t have one particular mandate, we can value add to whatever it is… (Manager)

Respecting the knowledge of the workers ‘on the ground’ who are discerning about community needs and supporting their initiative was also seen as important:

…as much as those agency workers want to step outside what they can do, and want to work with other agencies to come up with a project that meets the needs of [a] particular community, they can’t get the money to do it. (Administrator)

#### Coordination and collaboration

It is no coincidence that each of the case study initiatives was structured as an inter-sectoral partnership, reflecting both elements of social capital theory and principles of health promotion. An emphasis on connection and networks as important resources clearly leads to structures which reinforce the joint responsibilities of government agencies rather than a ‘silo’ approach. Similarly, advancing health and wellbeing at the community level is known to rely not only on the provision of health services, but also on the contributions of sectors like housing, urban planning, education and local government services [1,24]. Hence collaboration and partnerships across organisations were seen to be essential elements in a social capital approach to health equity.

Ideally you would have local government and planning and housing and so on all working together… (Administrator)

I think they [community based initiatives] can be very good value for money, particularly if you aggregate health, justice, child welfare and educational outcomes…. (Administrator)

…we’re a housing authority so to… spend our housing dollars on health then we get less housing outcomes. We don’t want to do that, but what we do want to do is link in with other government departments who have health as a role, and look at ways where we can undertake joint projects. (Administrator)

Nevertheless all respondents acknowledged that achieving effective partnerships can be challenging:

So it’s all very well to use the rhetoric but when they go back into Adelaide and say ‘we need funding to do this’, they say ‘well you can’t. That’s a DECS [Department of Education and Children’s Services] responsibility’. And DECS will sit there and say ‘well that’s not our responsibility, that’s Housing SA’s responsibility’. It’s just not happening. [So] from a practical perspective, nothing’s changed. (Administrator)

Every agency has different philosophies that can become a barrier to working together… (Administrator)

In one case study this issue had been addressed from the outset by building partnership development into the program plan and specifying it as an expected outcome in the subsequent evaluation. In this instance six sectors (health, education, housing, arts, local government and private land developer) were seated around the decision making table for the three-year life of the public art initiative. This partnership structure was a condition of funding, indicating recognition at the policy level that the quality of governance relationships can determine the success of the initiative:

That was the intention of the funding…to learn from each other… (Practitioner)

The fact that you can seat six managers down and make some collective decisions around arts [projects]… (Manager)

#### Building and utilising knowledge

In addition to the need for clarity of purpose and some understanding of social capital theory identified by the senior managers above, knowledge emerged as a theme in two main ways in the study. Firstly, there was repeated demand for support in terms of measuring social capital outcomes. Credible measures were judged to be critical in demonstrating the effectiveness of this approach to funding bodies, and contributing to a body of evidence for the application of social capital theory. Practitioners were often confident about being able to evaluate the activities at the local level in terms of short term effects on participants, but lacked precise means of linking these to particular elements of social capital and health outcomes.

I guess what would be helpful is ideas of measurement… so that there is a way of perhaps being more intentional about the stages of thinking. (Practitioner)

…that is always something that we could do better…actually take more time to do evaluation and to think about what we are really doing… (Practitioner)

Secondly, the role of the community worker included being local ‘interpreter’ , building and disseminating knowledge. This involved translating abstract ideas about social capital into practical language and activities at the local level, as well as transmitting local knowledge about needs, issues and effective strategies to decision makers in order to inform program development.

### Advocacy for ‘a social capital approach’

Disseminating findings to a wide audience in a form accessible and useful to practitioners and policy makers was a central aim of the study from its inception. In recognition of the potential benefits to health and wellbeing as well as the difficulties of operationalising complex concepts like social capital, the project sought to develop a resource to support good practice based on the knowledge gained. Steps in this process were informed by consultation with the Reference Group. In the first instance a working definition of social capital was developed which clarified its reference to ‘the connections, trust and reciprocity between individuals and within communities, and the resources that can arise from these connections… [including] employment or educational opportunities for individuals, and cohesion and a sense of safety in communities.’ The findings from case studies outlined above enabled identification of particular barriers to effective implementation as well as the lessons learned from practical experience. Reference Group members and other government administrators contributed to the analysis of these findings and helped to refine the concept of a ‘social capital approach’ through their attendance at workshops and ‘Think Tanks’. In accordance with the project aims, this approach was outlined in the development of the handbook ‘Practical Social Capital: a Guide to Creating Health and Wellbeing’ [25].

In addition, the Reference Group identified a need for a different kind of resource directed at the level of policy makers. As well as local factors such as community development skills and experience, the case studies had pointed to upstream factors in the policy environment which determined whether community programs could flourish or founder. The Reference Group similarly stressed the importance of gaining the imprimatur of decision makers at the highest levels of government to support implementation. They therefore recommended the development of an advocacy tool which would distil the study findings for this audience and convey both the advantages of a social capital approach in terms of policy outcomes as well as their own role in achieving such outcomes. This led to the publication of a companion volume directed at the more senior managers and policy makers titled: ‘Practical Social Capital: A Policy Briefing’ [26]. A key part of this document is the synthesis of the study findings in the form of a framework depicting a social capital approach as a matrix of mutually supportive relationships. In this integrated framework the actors and structures at the policy level are seen to influence directly what is achievable at the community level. The framework for ‘a social capital approach’ is presented in Table 2 below.

**Table 2:** A social capital approach in practice

**For policy & decision makers**	**For local practitioners**	**For communities & individuals**
Policies and frameworks guide and enable effective practice at the community level	Skilled and well-supported community workers facilitate community development and local initiatives	Individuals and groups in communities develop strong networks of mutual support and social action and gain economic and other resources as a result
**Long term vision & commitment**		
• Focus on social and economic sustainability despite demands of short term political agendas	• Base initiatives on analysis of changing needs and developmental approaches	• Experience long term changes with positive impacts on health and wellbeing
• Endorse community capacity building and development as valued strategy across government	• Develop strategies for meaningful change rather than ‘quick fix’ with superficial impact	• People in communities are part of the solution instead of being seen as part of the problem
• Invest in the future through planned on-going programs rather than short term ‘projectism’	• Link small and manageable local initiatives into coherent programs through coordinated planning	• See lasting positive changes occur through public funding
• Explicit policy statements on health equity		• Community people come to recognize that meaningful change can happen
**Sectors working together**		
• High level endorsement of importance of collaboration	• Pool resources to achieve better outcomes through effective and efficient collaboration	• Services and initiatives are better able to respond to people’s daily lives with consistency and coherence
• Integrate programs across sectors to avoid the ‘silo’ effect	• Underpin projects and programs with long-term social & economic goals as well as short term milestones	• Economic objectives support the achievement of equity and population health
• All sectors committed to social& health equity	• Reward workers for working together to generate more effective ideas and share resources	• Provision of support services such as transport and childcare improves access to services
• Make collaborative and cooperative ventures standard practice		
**Building effective relationships**		
• Provide incentives for programs and funding frameworks for community building and participatory approaches	• Engage local people in developing positive strategies as a priority	• Local people have good reason to become involved and stay engaged
• Support resources and training to develop healthy and long term relationships	• Take time and care to ensure that involvement is democratic and relationships are respectful	• Relationships are built on trust and respect for others’ roles and contributions
	• Make workers feel supported and rewarded for taking on the complex and demanding work of community development	• People have access to a variety of helpful support networks and social relationships (bonding, bridging & linking social capital)
	• Attract and retain experienced and skilled staff to community development projects	
**Generating knowledge about what works**		
• Learn about complex models of change from national and international experience	• Design projects using what is known about models of effective practice	• Best use of available time and resources
• Provide support and resources for monitoring and evaluating change as it occurs	• Ensure skills and resources are available to assist in gathering information about what works and why in the local context	• Evaluation is directed towards learning and improving and based on an understanding of complex models of change
• Respect different kinds of knowledge and expertise	• Collect valuable knowledge at the local level from workers and community members and use it to improve practice	• People feel valued and able to work as partners in developing ideas and strategies for improving health and reducing inequities

Based on the study findings, Table 2 is structured around the key factors that emerged as practical lessons from the case studies outlined above: ‘long term vision and commitment’ , ‘sectors working together’ , ‘building effective relationships’ and ‘generating knowledge about what works’. It then shows how factors such as ‘investment and support from state agencies’ and a ‘broader public policy environment committed to social equity’ prepare the way for the impact of local community development initiatives. The latter, in turn, fosters communities which are more engaged and better able to contribute towards achieving higher level government policies and goals related to health, wellbeing and equity. In this way, a social capital approach emerging from this study draws attention to the connections between actors at each level and highlights their equal contribution.

Limitations in the study should be noted in relation to the above presentation of results and framework. The recruitment of staff, managers and policy actors responsible for the selected cases as study participants and in some cases co-investigators potentially creates a conflict of interest. Their investment in the programs increases the likelihood that they will unwittingly cast the programs in a particular, positive light. This is ameliorated somewhat by the fact that the paper is not concerned with an evaluation of the effectiveness of these programs but with how they represent a particular interpretation of social capital concept and theories. Nevertheless, while the dual role of these key informants embodies a strength insofar as it offers a detailed understanding of the context and ensures the transfer of knowledge generated into the policy and practice realm, their views could also be regarded as partial. Our framework emerged as a result of the iterative methodology involving Industry Partners. Although suggestive of wider application, there is a need for the framework outlined in Table 2 ‘A social Capital approach in practice’ to be tested more explicitly, firstly in the Australian research context and then in other comparable settings.

## Conclusions

As discussed above the past decade has witnessed a growing recognition of the influence of social determinants of health [1]. In this context, linkages between social capital measures and health and wellbeing have been identified across macro, meso and micro levels, in the form of bonding, bridging and linking networks [13,14,27]. Interventions at the community level in particular aimed at strengthening social supports and networks have been shown to create important resources for health [9]. Given that community based initiatives based on social capital theory promise to contribute significantly to the reduction of health inequities by influencing community cohesion and individual resources, it is important to pay attention to the clarity of translating theory into practice and the quality of implementation. This collaborative study has shown that there was considerable interest among South Australian policy makers, administrators, managers and practitioners in the potential for social capital theory to inform community initiatives designed to promote health and wellbeing. It reinforced the need for greater theoretical clarity and accessibility at all levels, however, in order to realise this potential.

The case studies indicated that social capital in practice shares a symbiotic relationship with concepts like ‘community development’ and ‘capacity building’ [3,28]. While the trust and networks associated with social capital have been regarded as pre-requisites for community development strategies, this study suggests that community development can also contribute to stronger social capital. Although the findings highlighted the importance of community development techniques, in drawing attention to the interdependence between levels of decision making and action – policy, practice and community – they also underlined the joint responsibility across all levels of government for the successful application of initiatives at the local level. In short, community development approaches have far more potential to be sustained and effective and therefore greater potential to contribute to equitable health and wellbeing outcomes if they are supported at all levels of government policy and program management.

## Competing interests

The authors have no competing interests.

## Authors’ contributions

CP contributed to the design and management of the study, analysis of the results and prepared the preliminary draft of the manuscript. FB played a leading part in the design and management of the study and contributed to the analysis of the results and preparation of the manuscript. AZ contributed to the design and management of the study, analysis of the results and preparation of the manuscript. KA contributed to the design and management of the study, analysis of the results and preparation of the manuscript. DP coordinated the study, played the major role in the collection and analysis of data and drafted an earlier version of the manuscript. All authors approved final manuscript content.

## Pre-publication history

The pre-publication history for this paper can be accessed here:

http://www.biomedcentral.com/1471-2458/13/517/prepub
